# Bioactive Molecules for Skin Repair and Regeneration: Progress and Perspectives

**DOI:** 10.1155/2019/6789823

**Published:** 2019-12-31

**Authors:** Deyun Chen, Qian Hou, Lingzhi Zhong, Yali Zhao, Meirong Li, Xiaobing Fu

**Affiliations:** ^1^Wound Healing and Cell Biology Laboratory, Institute of Basic Medical Science, Chinese PLA General Hospital, Beijing 100853, China; ^2^Trauma Treatment Center, Central Laboratory, Chinese PLA General Hospital, Hainan Branch, 9 Haitang Bay, Sanya 572014, China

## Abstract

Skin regeneration is a vexing problem in the field of regenerative medicine. A bioactive molecule-based strategy has been frequently used in skin wound healing in recent years. Bioactive molecules are practical tools for regulating cellular processes and have been applied to control cellular differentiation, dedifferentiation, and reprogramming. In this review, we focus on recent progress in the use of bioactive molecules in skin regenerative medicine, by which desired cell types can be generated *in vitro* for cell therapy and conventional therapeutics can be developed to repair and regenerate skin *in vivo* through activation of the endogenous repairing potential. We further prospect that the bioactive molecule-base method might be one of the promising strategies to achieve *in situ* skin regeneration in the future.

## 1. Introduction

The skin is the largest organ in the body and plays a crucial role in protecting the body from various injuries, such as trauma, heat, chemicals, UV radiation, and microbial infection [1]. However, when the skin is injured, its protective function is lost, and the defects regrow new skin through a wound healing process. This process can be divided into three individual but overlapping phases: inflammation, reepithelialization, and tissue remodeling. It is a well-coordinated process involving a variety of cell types, mainly including immune cells, keratinocytes, fibroblasts, endothelial cells, and hair follicle stem cells [2]. Keratinocytes migrate to the wound site through proliferation and differentiation until the wound is entirely sealed [3]. Fibroblasts are the predominant cell type during the early stages of the wound healing process. A large number of the native fibroblasts transform into myofibroblasts, which are responsible for wound contraction and extracellular matrix (ECM) deposition [4, 5]. In addition, the reconstruction of an injured skin vascular network through the migration and proliferation of endothelial cells is necessary for successful wound healing [6]. The skin includes a large number of appendages, such as hair follicles and sweat glands. Hair follicle stem cells (HFSCs) are currently thought to be essential for hair follicle regeneration and skin repair, including differentiation into epidermal cells, sebaceous gland cells, and different types of hair follicle epithelial cells [7]. Moreover, sweat gland cells are responsible for the regulation of body temperature and contribute significantly to skin repair, presenting a substantial turnover both in wound healing and in homeostasis [8]. More importantly, these cells cooperate to repair/regenerate the injured skin, and abnormal function or an insufficient number of repairing cells frequently lead to scar healing or chronic wound.

Realizing skin regeneration is a worldwide problem. We propose to focus on two pivotal aspects: first is replenishing the sufficient number of repairing cells and second is activating the endogenous repair potential. Therefore, cell transplantation, skin grafts, and tissue-engineered skins are commonly used for skin wound healing. For example, one study illustrated the use of keratinocytes and fibroblasts suspended in the platelet-rich plasma-enriched medium which could promote the full-thickness skin wound healing [9]. Another *in vivo* study showed that bacterial cellulose/acrylic acid hydrogel loaded with human epidermal keratinocytes and dermal fibroblasts leads to the higher acceleration of burn wound healing, compared with treatment with hydrogel alone [10]. A recent study reported a compound biomaterial which is constructed with nanofibrous collagen, polycaprolactone, and bioactive glass nanoparticles which promoted the proliferation, migration, and vascularization of endothelial progenitor cells through upregulation of the hypoxia-inducible factor-1*α*/vascular endothelial growth factor/stromal cell-derived factor-1*α* (HIF-1*α*/VEGF/SDF-1*α*) signaling pathway [11]. All of these studies illustrated the importance of supplemental necessary repairing cells for wound repair. Still, it is worth noting that the lack of seed cells is an outstanding limitation for the widespread adaptation of the strategy. Recently, stem cells, mainly induced pluripotent stem cells (iPSCs), have received much attention for their tissue repair and regeneration properties. They can provide unlimited required cells and are theoretically able to differentiate into all cell types in the body, which are considered an ideal cell source. More importantly, iPSCs have the advantage in autotransplantation, which can avoid immune rejection. Nevertheless, many studies have demonstrated that genetic approaches are the conventional methods for cell reprogramming or transdifferentiation, which is low efficiency and has the integration risk. Therefore, it cannot be used as a conventional strategy for getting target cells for cell therapy [12]. Thus, compared to genetic manipulation, bioactive molecule-based reprogramming with high efficiency and improved quality of the reprogrammed cells is one of the critical approaches for obtaining cells of interest.

**Figure 1:**
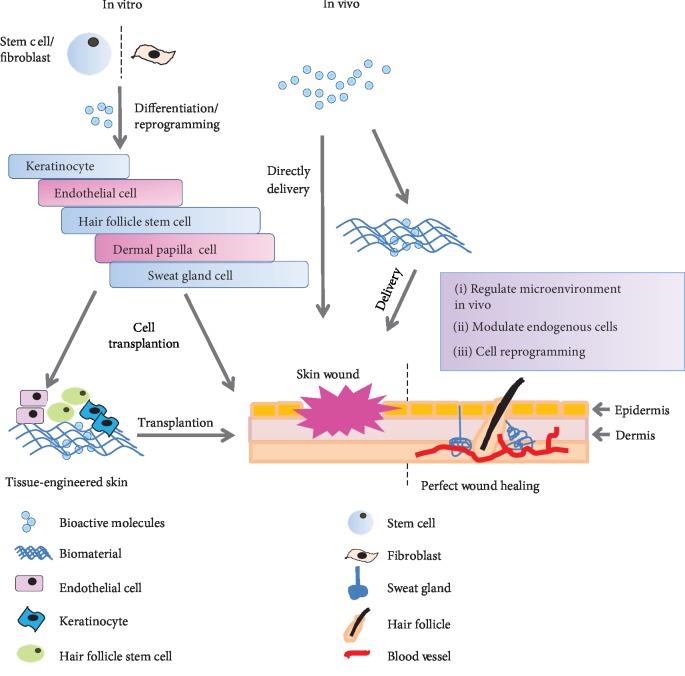
The strategies of skin regeneration using bioactive molecules. The repairing of cells induced from stem cells or somatic cells by using bioactive molecules *in vitro* for skin repair or stimulating skin endogenous cells to regenerate skin in vivo by using bioactive molecules as a conventional therapeutics.

The bioactive molecules mentioned here include large natural molecules, such as proteins, cytokines, and lipids, as well as small molecules that have been demonstrated to regulate specific signaling pathways and contribute to cell reprogramming and tissue repair. Bioactive molecules are mostly cell-permeable and nonimmunogenic and can selectively modulate intracellular processes in a reversible way with their cellular targets [13, 14]. Therefore, bioactive molecules with therapeutic potentials may represent the next generation of regenerative medicine. More importantly, bioactive molecules have been used for cell differentiation or reprogramming to acquire the target cells with biological activity *in vitro* [15–17]. For example, human pluripotent stem cells (hPSCs) generated mesodermal cells after treatment with CHIR99021 and bone morphogenetic protein4 (BMP4). Mesodermal progenitors differentiated into vascular endothelial cells in the exposure to vascular endothelial growth factor A (VEGF-A) and the small molecule forskolin *in vitro*. And the hPSC-derived endothelial cells could be further cultured and expand in medium supplemented with serum, VEGF, and SB431542 [18]. Additionally, not only can bioactive molecules promote reprogramming and differentiation efficiency, but they can also activate the local repair potential. The C-X-C chemokine receptor type 4-stromal cell-derived factor 1 (CXCR4-SDF1) axis plays a vital role in the stem or progenitor cell mobilization and home, which can be modulated through bioactive molecules. For example, antagonizing CXCR4 with Plerixafor/Mozobil induced stem cell or progenitor cell mobilization, while increasing CXCR4 expression with prostaglandin E2 or enhancing SDF1 stability with Diprotin A promoted stem cell or progenitor cell homing [19]. Therefore, it is conceivable that bioactive molecules can be developed as a treatment, which can be delivered *in vivo* directly to repair injured tissues and regenerate damaged or lost cells. This review will focus on the recent developments of bioactive molecules that contribute to skin wound healing. We emphasize on the repairing cells reprogrammed from other cells through bioactive molecules' induction and the endogenous repairing cells recruited from local and distant tissue by bioactive molecules' stimulation (Figure 1).

## 2. Skin-Repairing Cells That Are Induced by Bioactive Molecules

### 2.1. Keratinocytes Derived by Bioactive Molecule Induction

Keratinocytes make up the first barrier of the skin. They play a critical role in the reepithelialization process which is mediated by keratinocyte proliferation and migration. If this reepithelialization process failed, its barrier function is lost, which might cause dehydration, infection, or even death [20, 21]. Rapid reepithelialization is indispensable for restoring the skin barrier. Keratinocytes can be used as grafts or as a component of other complex matrices to cover the injured sites [22]. Nevertheless, keratinocyte sources are limited. It is necessary to develop new strategies to obtain sufficient keratinocytes for skin wound transplantation.

Employing bioactive molecules to modulate signaling pathways of keratinocyte development and to restrict initial cell to follow the specific differentiation path presents an alternative strategy for obtaining transplantable keratinocytes. Previous studies showed that the transgene method was commonly used for cell reprogramming, while the risk of insertional mutagenesis of viral vectors and spontaneous transgene reactivation limit its application in the clinic [23]. Reprogramming and transdifferentiation through bioactive molecules have been explored and tested in regenerative medicine in recent years. Primarily, iPSCs were initially generated by viral vector-mediated overexpression of pluripotency factors (Oct4, Sox2, Klf4, and c-Myc (OSKM)) from fibroblasts. Then the pluripotency factors were substituted by a combination of bioactive molecules [24]. A large number of studies show promise in obtaining keratinocytes by bioactive molecules' strategy from embryonic stem cells (ESCs) and induced pluripotent stem cells (iPSCs). And in this process, the bioactive molecule selection is a vital prerequisite that depends on an understanding of keratinocyte development and regulatory mechanism, as well as further screening the suitable bioactive molecules to guide initial cell reprogramming into keratinocytes.

In the skin development, the epidermis is derived from the primitive ectoderm, which expressed K8/K18, and then these cells committed to a keratinocyte fate which was marked by the K5/K14 expression replaced by K8/K18 expression. The K5/K14-positive basal keratinocytes initiated epidermal stratification and eventually underwent terminal differentiation to form the mature adult epidermis marked K1/K10 [25], while the primitive ectoderm also gives rise to the nervous system and its choice to epidermal lineage development is governed by coordination of Wnt, bone morphogenetic protein (BMP), and fibroblast growth factor (FGF) signaling. Among them, Wnt signaling was active in early epidermal progenitors and promoted ectodermal cells to differentiate into epidermal [26, 27]. With Wnt signals, the ectodermal cells responded to BMP signaling while inhibiting FGF signaling, which resulted in an epidermal fate. Without Wnt signals, the ectodermal cells responded to FGF signals instead of BMP signaling activity and thus developed towards a neural fate [28, 29]. Also, p63, a transcription factor, which is required for initiating a basal layer of keratinocytes, was expressed throughout the epidermal differentiation [17]. BMP signaling also has been suggested to control p63 expression during ectodermal development. *ΔNp63* is an ectoderm-specific direct transcriptional target of BMP signaling, which is essential for the dorsoventral patterning of the ectoderm and skin development. The early expression of *ΔNp63* in the ectoderm is directly activated by Smad4/5-mediated BMP signaling and is sufficient to block neural specification while promoting the early steps of the epidermal specification [30]. Moreover, *ΔNp63* also can directly induce K14 expression [31].

According to the roles of crucial regulators and signaling pathways during embryonic epidermal development, pluripotent stem cells (PSCs), including ESCs and iPSCs, can differentiate into keratinocytes by sequentially activating the above signaling pathways. Based on previous studies, BMP4 and retinoic acid (RA) have been used as well-known inducers for epidermal differentiation from ESCs *in vitro* [32, 33]. *In vivo*, BMP4 is a potent epidermal inducer and a neural inhibitor along with ectodermal fate [34]. During the differentiation of ESC into epidermal cells in vitro, BMP4 activates the expression of *ΔNp63*, further promoting expression of basal keratinocyte maker K5/14 and the terminal differentiation marker K1/10 expression [26]. BMP4 also could promote epidermal commitment by downregulating Smad6 and induce the apoptosis of Sox1+ neural progenitors [35]. Besides, BMP4 upregulated ectoderm protein AP2*γ* (a transcription factor activator protein- (AP-) 2 family member) through binding of Smad1 to its promoter, which could restrict neural expansion and initiate epidermal differentiation during the early stages of ectodermal patterning [36]. RA was also discovered as an inducer of keratinocyte differentiation from iPSCs or ESCs. RA was a stage-dependent agent for differentiation which directed pluripotent stem cells differentiating into ectodermal cells and upregulated K8/18 and p63 expression [37]. Therefore, RA and BMP4 play distinct but synergistic roles in promoting iPSC or ESC differentiation along the keratinocyte lineage and inhibit neural differentiation [32, 38]. Additionally, studies showed that BMP4 and RA promoted the differentiation of pluripotent stem cells into the epidermal through inhibition of the canonical Wnt signaling pathway. RA could induce the localization of *β*-catenin to the cell membrane and diminished the amount of nuclear *β*-catenin, which downregulated the canonical Wnt signaling and activated the noncanonical Wnt signaling pathway [39]. Therefore, the downregulation of canonical Wnt signaling might be able to induce the epidermal differentiation of pluripotent stem cells. SU6656, a Src family kinase inhibitor, has been shown to modulate *β*-catenin translocation to the cell membrane and directly phosphorylate *β*-catenin; this phosphorylation inhibits the binding of *β*-catenin to E-cadherin, which suppresses the expression of the canonical Wnt-dependent genes. SU6656 induced the differentiation of hPSCs into epithelial cells with the repression of pluripotency gene Oct4 and upregulation of ectoderm marker K18/K8. These hPSC-derived keratinocytes possessed the capacity to terminally differentiate, which were capable of forming coherent epithelial sheets or could be used for skin tissue engineering applications [40, 41]. These keratinocytes provide an abundant source for cellular transplantation applications and regenerative medicine [17, 42]. Also, keratinocytes can be derived from the two-step differentiation process of iPSCs. Recombinant noggin, SB431542, and CHIR99021 created human skin-derived precursor cells from human-induced pluripotent stem cells (hiPSCs) that could differentiate into keratinocyte-like cells on a collagen-chitosan scaffold, which contributes to the regeneration of the epidermal and dermal layers [43]. As mentioned in this section, the iPSC-derived keratinocytes that were induced by the above bioactive molecules have similar biological functions as the keratinocytes in the physiological state [44].

It is worth noting that according to the above differentiation pathway, we need to obtain PSCs first and then further induce the differentiation of PSCs into physiological keratinocytes. Fortunately, the “two-step differentiation process” controlled by bioactive molecules is now available. However, such a reprogramming path for getting keratinocytes will elevate the risk of the accumulation of cellular damage, even tumor formation. Therefore, how to directly reprogram adult stem cells, even the reprogramming of other somatic cells into keratinocytes, is worth exploring.

Direct cellular reprogramming is often dependent on the substitution of key transcription factors. One study has shown the direct conversion of mouse embryonic fibroblasts into functional keratinocytes, in which the fibroblasts transfected with Sox2, Oct4, and Klf4 to initiate part dedifferentiation and then are treated with RA and BMP4 for their differentiation [45]. Another study has reported that the combination of the transcription factors p63 and Klf4 can convert human fibroblasts to keratinocyte-like cells [46]. Unfortunately, these studies still use genetically modified methods. If bioactive molecules replace these critical transcriptional factors, it will achieve the goal of getting keratinocytes through direct reprogramming by bioactive molecules, which is a promising method to get unlimited keratinocytes for cellular transplantation and tissue-engineered skin. The latest research reported that simvastatin could differentiate HFSCs into keratinocyte. Besides, simvastatin accelerated the wound healing through anti-inflammatory, anti-bacterial, immunomodulatory, and antioxidative effects and the improvement of vascularization [47]. Therefore, bioactive molecules with the dual forces of promoting the orientation-inducing ability and promoting the repairing fact are more favored.

### 2.2. Endothelial Cells Derived by Bioactive Molecule Induction

Revascularization is an essential stage of the wound healing which establishes blood supply to the newly formed tissue. New blood vessels provide the nutrients and oxygen for the newly formed granulation tissue [48]. Endothelial cells and their progenitors are essential in neovascularization [49, 50]. Moreover, endothelial cells have two primary functions for tissue homeostasis. The first one is to release the tissue-specific angiocrine factors that support tissue homeostasis and regeneration, and the second is to regulate the permeability of proteins, ions, bioactive molecules, and even cells through the vascular wall impacting on a trauma microenvironment [51]. Similar to the strategy of obtaining keratinocytes, endothelial cells can be obtained by PSC differentiation or by direct reprogramming of fibroblasts. These induced endothelial cells and their progenitors may provide the cell source for the construction of tissue-engineered vascularization. To support these applications, an efficient and reliable *in vitro* differentiation induction system is needed. The differentiation method *in vitro* should follow the process of endothelial cell development, and the cultural environment *in vitro* should simulate the microenvironment of the target cells *in vi*vo. Endothelial cells are derived from mesodermal progenitors during embryonic development. Activin/Nodal/TGF-*β*, BMP, Wnt, and fibroblast growth factor (FGF) signaling pathways play crucial roles in the mesodermal transition during gastrulation and are required for epiblast-to-mesoderm transition. Therefore, Activin A/Nodal, BMP4, FGF2, and Wnt ligands or GSK3 inhibitors (such as CHIR99021) were currently the most used mesoderm-endothelial cell inductive factors *in vitro* [52–54]. Modulation of the signaling as mentioned above should promote the differentiation of PSCs into functional endothelial cells.

So far, there are three methods to differentiate PSCs into endothelial cells: coculture of PSCs with stromal cell lines, three-dimensional (3D) embryoid body- (EB-) mediated differentiation, and monolayer-directed differentiation. The latter two methods are accomplished by using bioactive molecules to follow the developmental process. PSCs differentiated into mesodermal lineages and then differentiated into endothelial cells under the treatment with Activin A, BMP4, FGF2, VEGF-A, SB431542, and so on [48, 55–57]. And it was reported that hESCs aggregated into EBs, and then sequential exposure to BMP4, Activin A, FGF2, VEGF-A, and SB431542 drives the differentiation of the cells into endothelial cells which were functional in *vivo.* And the SB431542 was the critical small molecule for enhancing cell differentiation efficiency, up to tenfold [58]. In line with the above study, recent studies also have shown that the inhibition of TGF-*β* using SB431542 enhances the production of endothelial cells from hPSCs, which may be due to SB431542 resisting the inhibitory effect of TGF-*β* on endothelial expansion [59, 60]. The latter study showed that activation of canonical Wnt signaling via CHIR99021 without exogenous growth factors was sufficient to generate high yields of endothelial progenitors from hPSCs [61–63]. The other study demonstrated that hPSCs differentiated into endothelial cells in the exposure to CHIR99021, and these cells generated intact microvessels *in vitro* in PDMS-based microfluidic devices, which could be subjected to shear stress to mimic the in *vivo* physiological conditions [64]. And the promotion effect of CHIR99021 was by the upregulation of CDX/HOX genes, which are expressed in the posterior mesoderm of embryo development [65]. These *in vitro* differentiation experiments also confirmed the importance of activation of the Wnt signaling pathway for generating mesodermal progenitor cells from PSCs, and these cells could be further differentiated into vascular endothelial cells or cardiomyocytes. Of course, the proper combination of bioactive molecules further improved the differentiation efficiency. For example, high differentiation efficiency was achieved when CHIR99021 was combined with a lower concentration of VEGF-A and DLL4 (a Notch ligand) in the endothelial cell induction system [66]. DLL4 could enhance endothelial cell-lineage differentiation and inhibit hematopoietic-lineage differentiation [67]. In addition, CHIR99021 in combination with FGF2, VEGF, and BMP4 could synergistically induce early vascular progenitors (VPs) from hiPSCs with high purities. It is established that VEGF and FGF2 activated the MAPK and the PI3K pathways, which were crucial not only for the initial commitment to vascular lineages but also for the differentiation of vascular progenitors to endothelial cells. It most likely through regulation of the ETS family transcription factors, ERG and FLI1 which were important to endothelial cell development [68]. Except for growth factors and small molecules, 3D culture also enhanced differentiation efficiency; the 3D thermoreversible PNIPAAm-PEG hydrogel provided 3D space for cell growth and acted as a physical barrier to prevent the cell from agglomeration and the shear force during the differentiation of endothelial cell [69]. More importantly, iPSC- or ESC-derived endothelial cells are capable of forming capillary-like structures *in vitro* and integrating into the host vasculature *in vivo* [18]. These studies have proven the possibility of generating intact, engineered microvessels *in vitro* that replicate some of the fundamental biological features of native microvessels.

Direct reprogramming is another way to obtain endothelial cells. Endothelial cells are derived by direct reprogramming from fibroblasts, and amniotic cells have also been reported [70, 71]. Human fibroblasts could be transdifferentiated into endothelial progenitor cells using lentiviral ETS transcription factors (ETV2) and hypoxia, the derived endothelial progenitor cells similared to human umbilical endothelial cells in phenotypes and tube formation assay [71, 72]. We have mentioned that the use of lentiviral vectors raises safety issues as it may induce insertional mutagenesis, as well as possible generation of replication-competent lentiviruses and germline transfer. Furthermore, the efficiency of the direct reprogramming of lentiviral vectors is low. Therefore, bioactive molecules that can replace critical transcriptional factors may be a feasible strategy for direct reprogramming. It has been proven that with the sequential addition of 5-aza-2′-deoxycytidine and trichostatin A in a fibroblast culture environment and tideglusib for a specific time, the fibroblasts can convert into functional endothelial progenitors. 5-Aza-2′-deoxycytidine, a DNA methyltransferase inhibitor, and trichostatin A, a histone deacetylase inhibitor, may cause chromatin decondensation and induce a short “dedifferentiation” state. Tideglusib inhibited *β*-catenin phosphorylation, which resulted in its translocation to the nucleus activation of Wnt signaling, thus causing the differentiation [73]. These results illustrate the feasibility of direct reprogramming for obtaining endothelial cells by bioactive molecule induction, but more achievable paths and the critical signaling pathways for reprogramming need to be further explored.

### 2.3. Hair Follicle Cells Derived by Bioactive Molecule Induction

Hair follicle (HF) undergoes cyclical physiologic regeneration, and their cellular components exhibit robust regenerative capabilities. Hair follicle stem cells (HFSCs) residing in the bulge represent a unique source of stem cells that are maintained through self-renewal and differentiation during the hair cycle and contribute to hair follicle regeneration and wound healing [74]. Following wounding, skin wound healing started not just from the edges of the wound but also around the follicles. Hair follicle-derived epidermal stem cells (EpSCs) are recruited immediately for wound closure. It has been shown that skin injury in high hair density areas tends to heal more quickly than those lacking hair follicles. And a chronic wound treated with skin grafts with hair follicles showed a faster healing rate than that transplanted with skin grafts without hair follicles [75].

Hair follicle morphogenesis needs epithelial-mesenchymal interaction and involves many signaling pathways, such as Wnt, BMP, Shh, Notch, TGF-*β*, and platelet-derived growth factor (PDGF) [76–79]. For example, Wnt signaling is necessary for hair follicle development and patterning, and Wnt ligands are expressed in the interfollicular epidermis as well as the hair follicle during all stages of follicle development [80]. In the process of epidermis development, a high level of Wnt signaling is essential for HF induction, while the constitutive attenuation of epidermal Wnt signaling impairs HF formation, but does not impact interfollicular epidermis integrity. Deletion of *β*-catenin in the skin epidermis or overexpression of Dkk1, a soluble inhibitor of Wnt signaling, results in the absence of HF with no sign of HF or dermal papilla (DP) formation [81]. BMP signaling has also been suggested to regulate hair follicle induction and the patterning of follicles in the skin [79]. In the mature HF, the quiescence and activation of HFSCs are tightly controlled by a balance of BMP and Wnt signals coming from their niche cells. Decreased BMP signaling unleashes Wnt signaling activation promoting HF growth. In early epidermal development, Shh is expressed in the placode of developing HFs. Shh is required for hair follicle morphogenesis during embryogenesis and for regulating follicular growth and cycling in adults. While lacking *β*-catenin in the epidermis, Shh is not expressed, which indicates that Shh signaling serves as a downstream pathway of Wnt/*β*-catenin signaling to regulate HF induction. Therefore, the development and circulation of hair follicles need the various signal pathways to interconnect and mutually constrain.

At present, there are a few reports on the stem cell-derived EpSCs. Some results showed that BMP and Wnt are necessary for maintaining EpSC features during HF formation in the embryo and adults. BMP signaling is essential for hair cycling, and Wnt signaling pathway activation is required to stimulate hair growth [82]. One study has shown that hiPSCs differentiate into EpSCs through precise temporal control of the activities of epidermal growth factor, RA, and BMP4. These EpSCs can reconstitute the epithelial components of the hair follicle and interfollicular epidermis [83]. And the latest study produced EpSCs from iPSCs using the same method as mentioned above with minor modifications; iPSC-derived EpSCs seeding on the human acellular amniotic membrane (hAAM) promoted the construction of hair follicles and interfollicular epidermis and restored skin functions after transplantation [84]. IM176OUT05 activated HFSC metabolism and further promoted the expansion of ki67-positive progenitors during the early phases of hair follicle regeneration so that IM176OUT05 could be a candidate drug to improve tissue regeneration with high solubility, greater potency, and bioavailability [85].

Well-orchestrated epithelial-mesenchymal interactions are crucial for hair follicle morphogenesis [86]. In HF development, EpSCs are induced by mesenchymal dermal papilla (DP) cell cues to develop HFs. DP, located at the base of the hair follicle, plays a critical role in directing keratinocytes to form the follicle. The role of the DP in hair formation includes determining the size, shape, color of the hair, and the frequency with its regeneration [87]. Therefore, DP cells are important supporting cells for hair follicle regeneration after injury. Research on bioactive molecule-induced DP cells has also been frequently reported. A study has demonstrated that hiPSCs differentiate into induced mesenchymal cells (iMCs) with a bone marrow stromal cell phenotype and are subsequently induction with RA and DP cell culture medium to acquire DP properties. The resultant DP cells interacted with human keratinocytes to upregulate HF-related genes in keratinocytes and, when cografted with human keratinocytes *in vivo*, gave rise to a hair cuticle-like coat. However, the study also suggested that human DP cells lose key inductive properties after *in vitro* resemblance of the hair shaft [88]. This loss of inductivity was partially reversible if the cells are reassembled into three-dimensional (3D) spheroids [89]. Also, bioactive molecules play an essential role in maintaining the biological efficiency of DP cells. It showed that topical treatment of human skin with JAK inhibitor, tofacitinib, increased the growth rate of anagen hair shafts and enhanced the inductivity of human DP spheres [90]. Moreover, the bioactive molecule, 3-deoxysappanchalcone, an herbal medicine similar to the JAK inhibitor tofacitinib, promoted the proliferation of human hair follicle DP cells and hair growth via modulation of Wnt/*β*-catenin and STAT signaling [91].

In addition to the above critical cells, skin wound healing also requires sweat gland cells (SGCs) to help restore sweat glands (SGs). SGCs are responsible for the regulation of body temperature and are also critical for wound repair [8]. Like all skin appendages, sweat glands are derived from embryonic ectoderm. However, following severe skin injury, SGs do not regenerate because of the destruction of the duct and secretory cell coiler [92]. Up to now, SGCs are mainly obtained by environmental induction and the transgenic method. The previous study reported that human umbilical cord mesenchymal stem cells (hucMSCs) could differentiate into sweat gland-like cells under sweat gland cell-induction medium consisting of nine parts of basic SG medium and one part of sterile supernatants from a conditioned heat-shock SG medium, which might help solve the problem of sweat gland depletion in patients [93]. However, this differentiation rate was very low, and it was challenging to meet the needs of large-area transplantations. A later study showed that epimorphin could improve the rate of differentiation of mesenchymal stem cells into SGCs [94]. A recent research suggested that the human keratinocyte growth factor played a pivotal role in promoting hucMSC differentiation to sweat gland-like cells [95]. The first transgenic report transformed the key factors NF-kb and Lef-1 that were related to sweat gland development and directly reprogrammed fibroblasts into sweat gland-like cells [96]. However, no study reported on how to use bioactive molecules alone to obtain the SGCs, which needed further exploration. It is worth recognizing that the critical cell types required for wound repair can be obtained by bioactive molecule induction through the differentiation or direct reprogramming, which may provide a sufficient cell source for wound cell therapy.

It is not difficult to see that skin repair cells can be obtained using bioactive molecule-induced differentiation of stem cells, which is closely related to developmental science. The molecular mechanism explained from the developmental perspective is the essential information for realizing the differentiation of stem cells into target cells. Nevertheless, efforts still need to be made to more in-depth understanding of the developmental process of the skin to obtain a more effective induction strategy. In addition, we should further evaluate the role of repairing cells in tissue repair and the interaction between various cells, such as the hair follicle development mentioned above, and the microenvironmental signals provided by the supporting cells may be the key to tissue regeneration. Additionally, as far as cell therapy is concerned, the researchers tend to perform single-cell type transplantation. In contrast, the combined transplantation of different cells may achieve better therapeutic effects, which are also worth of attention. Even in the process of stem cell differentiation or direct reprogramming, it is also worth to pay attention to whether it is possible to obtain multiple skin cells in one induction system concurrently, thus promoting the regeneration of the skin by cotransplantation.

### 2.4. In Situ Skin Regeneration Is Induced by Bioactive Molecules

As we discussed above, it will require a large amount of expansion of target cells and takes a lot of time and money to obtain the functional repair cells for cell therapy. Therefore, is there a better way to promote tissue regeneration? Tissue repair requires sophisticated regulatory networks and specific manipulation of certain signaling pathways, while bioactive molecules may serve the purpose of recruiting stem cells, stem cell differentiation, transdifferentiation, etc. *in vivo*. So the direct application of bioactive molecules to the injured site is an important focus in tissue regeneration. Tissue regeneration stimulated with bioactive molecules in *vivo*, which is called *in situ* regeneration, has just emerged in recent years. For example, Thymosin 4 has been found to promote wound healing by decreasing inflammation, increasing angiogenesis, enhancing keratinocyte and endothelial cell migration, and promoting hair follicle growth [97]. Plerixafor in combination with G-CSF for hematopoietic stem cell (HSC) mobilization has been approved in multiple myeloma patients for autologous transplantation. Moreover, they showed that this HSC mobilization effect could be enhanced by BIO5192, a small molecule VLA-4 inhibitor [98, 99]. Another study showed that the sustained delivery of sphingolipid growth factor FTY720 from poly(lactic-co-glycolic acid) (PLAGA) and local targeting of sphingosine 1-phosphate receptors reduced infiltration of CD45+ inflammatory cell, promoted endogenous recruitment of CD29+CD90+ bone progenitor cells, and improved defective vascularization and bone formation [100]. A recent study demonstrated that a natural bioactive molecule from cruciferous vegetables, DIM (3,3′-diindolylmethane), could promote the stemness of hucMSCs, which provided a novel strategy for improving the therapeutic effects of hucMSCs on tissue repair [101]. Recently, *in vivo*, Tazarotene enhanced the growth of mature and functional microvessels in wound healing models, thereby accelerating wound neovascularization [102]. Hydroxysafflor yellow A and deferoxamine have been reported to accelerate wound healing through promoting angiogenesis and reducing the inflammatory. And the deferoxamine and hydroxysafflor yellow A hydrogel could accelerate diabetic wound healing via enhanced angiogenesis by upregulation of hypoxia-inducible factor-1 alpha expression [103]. AES16-2M, an extracellular signal-regulated kinase-activating peptide, activated the MAPK signaling pathway and promoted wound healing through accelerating the migration of keratinocytes [104]. Our latest research demonstrated that lithium chloride (LiCl) loaded into chitosan hydrogel in full-thickness loss showed reduced inflammation, improved angiogenesis, accelerated reepithelialization, and regenerated hair follicles [105]. Based on these above studies, the possible roles of bioactive molecules for regenerative medicine *in vivo* are through regulating the wound microenvironment, like reducing inflammation, increasing angiogenesis, modulating endogenous stem cell or progenitor cell proliferation, differentiation, and homing, stimulating somatic cell proliferation or repairing their function, and promoting somatic cell reprogramming into repairing cells.

Of course, these bioactive molecules can not only promote wound repair but also reduce scar formation and treat skin diseases. Inhibition of Wnt/*β*-catenin signaling has been proved to reduce fibrosis and promote regeneration of wounds. The application of small molecule, XAV-939, could reduce fibrosis and promote regenerative cutaneous wounds in response to inhibition of Wnt/*β*-catenin signaling [106]. ICG-001 inhibited the endogenous Wnt/*β*-catenin signaling and had an inhibitory effect on collagen production in fibroblasts, suggesting its potential application in fibrotic diseases [107]. Poly(*ε*-caprolactone)/gelatin (PGT) fibrous scaffold doped with the TGF-*β*1 inhibitor (SB525334) could be used for reducing scars [108]. Bacterial cellulose (BC) decorated with 4,6-diamino-2-pyrimidinethiol- (DAPT-) modified gold nanocomposites inhibited bacterial growth and promoted wound repair when applied as wound dressings [109]. Interfering with the JAK/STAT signaling by a small molecular pharmacological inhibitor (the STAT3 inhibitor STA-21) was currently feasible since it could be applied not only systematically but also topically for the treatment of inflammatory skin diseases [110]. Therefore, the use of bioactive molecules to reduce fibrosis and activate the endogenous repair potential can promote wound repair. However, the regeneration of skin wound appendages through the use of bioactive molecules is currently rarely reported. This may be due to the unclear molecular mechanism of appendage regeneration. With the more in-depth exploration of signaling pathways in regenerative skin biology, it is not difficult to efficiently obtain adequate targeted skin cells by bioactive molecules *in vitro*, and these bioactive molecules also might be used as drugs for skin repair and regeneration *in vivo*.

## 3. Perspectives

In this review, bioactive molecule-based reprogramming represents an attractive and valuable alternative approach to the repair/regeneration of skin wounds. Bioactive molecules induce stem cells to differentiate into keratinocytes, endothelial cells, hair follicle stem cells, DP cells, and so on, which may provide sufficient cell source for cell therapy or tissue engineering (Table 1). Moreover, direct reprogramming of somatic cells into skin cells through bioactive molecules by passing stem or progenitor cell status is also crucial for regenerative medicine, which simplifies the process and avoids the risks associated with using stem cells. Most protocols for generating target cells follow the embryonic development of the target cells through the stepwise of bioactive molecule combination induction. Bioactive molecules are stable, cost-effective, and cell-permeable, and they reduce the safety concerns about genetic manipulation and are becoming a traditional alternative in cell reprogramming and regenerative medicine [111, 112]. Nevertheless, there are still some challenges in using bioactive molecules as traditional treatment in regenerative skin medicine.

The first challenge is the low efficiency of chemical-based reprogramming or transdifferentiation. A better understanding of the detailed mechanisms during reprogramming or transdifferentiation processes can help to select more specific bioactive molecules to improve the efficiency. It is necessary to explore the molecular mechanisms and regulatory networks of skin development in temporal and spatial changes and further to screen for possible proregenerative bioactive molecule combinations which promote dedifferentiation of differentiated cells or transdifferentiation of one cell into another. These problems may be solved with joint efforts by developmental biologists, pharmacologists, and multidisciplinary experts.

The second challenge is to screen suitable bioactive molecules for skin repair in situ. The identification of bioactive molecules that can promote regeneration is primarily based on the *in vitro* cell model by high-throughput screening. Promoting skin wound healing through bioactive molecules *in vivo* represents a new direction. We propose that the functions of bioactive molecules mainly focus on the following four aspects: (1) recruit stem cells near and far from the skin wounds, such as HSCs, MSCs, and adipose stem cells, which have been proved to be useful in wound repair and regeneration [113]; (2) promote the dedifferentiation of keratinocytes, fibroblasts, and endothelial cells in order to increase the number of stem cells in skin; (3) regulate the inflammatory microenvironment of the wound, as it is well-known that excessive inflammation inhibits wound regeneration; and (4) increase the biological function of repair cells, such as to improve the ability of wound vascularization and the migration of repairing cells. For example, the bioactive molecule compounds consisting of alprostadil and trimebutine maleate promoted the self-renewal and proliferation of skin-derived precursor *in vitro* and enhance skin repair *in vivo* through the pharmacological activation of endogenous precursor cells [114]. As far as skin repair is concerned, we need to find more effective bioactive molecules that can target the activation, proliferation, differentiation, and survival of endogenous stem cells and further clarify their possible molecular mechanism.

The third challenge is that the *in vitro* results of bioactive molecules do not reflect the *in vivo* regenerative effects. How to efficiently deliver transplanted cells into the target tissues, to maintain their survival *in vivo*, and to engraft into the endogenous tissues still needs further study. Furthermore, verifying the regenerative effect of bioactive molecules *in vivo* requires many experiments *in vitro*, and we also need to pay attention to the consistency of the effects of bioactive molecules *in vivo* and *in vitro*; after all, the microenvironment *in vivo* is more complicated.

The fourth challenge is to devise a safe and efficient delivery system for transporting bioactive molecules into the target tissues. Because of the lack of specificity, more efforts should be made to reduce the off-target effects and evaluate the tumorigenic risk while using bioactive molecules to promote regeneration *in vivo* before clinical applications. Nevertheless, we hope that bioactive molecules together with current biological technologies will enable in situ skin regeneration and accelerate the progress in regenerative medicine. Scaffolds, hydrogel, or other biomaterials have been used as vehicles by providing controlled release of therapeutic agents and tunable release of bioactive molecules of skin engineering [115]. A study has shown that electrospun fibers can be bioactive molecule carriers for wound healing [116]. And a wide variety of bioactive molecules has been incorporated into electrospun fibers and delivered into injured sites. Recently, it has reported that a bifunctional hydrogel loaded with the sphingosine analogs, FTY720 and SDF-1*α*, enhances the recruitment of anti-inflammatory monocytes and promotes microvascular remodeling, thus promoting of tissue repair. This dual-affinity hydrogel overcomes the challenge of codelivering two physiochemically distinct molecules, and the corelease of FTY720 and SDF-1*α* yields superior proregenerative biological effects over either factor alone [117].

We propose that bioactive molecule-based tissue regeneration is not only an essential method in the field of skin repair but also an important strategy for the restoration of injured tissues in the future. With a better understanding of regenerative mechanisms in different tissues and the development of technologies for high-throughput screening, future studies in tissue regeneration by bioactive molecules will overcome current hurdles, generate discoveries, and benefit human health.

## Figures and Tables

**(a):** 

*In vitro*			
Initial cell	Target cell	Bioactive molecules	Reference
Pluripotent stem cells	Reprogramming to keratinocyte	BMP4, RA	[32, 33]
		SU6656	[17, 42]
		Noggin, SB431542, CHIR99021	[43]
Pluripotent stem cells	Reprogramming to endothelial cells	BMP4, Activin A, FGF2, VEGF-A, SB431542	[58, 59]
		CHIR99021	[61–64]
		CHIR99021, VEGF-A, DLL4	[66]
		CHIR99021, FGF2, VEGF, BMP4	[68]
Fibroblasts	Reprogramming to endothelial cells	5-Aza-2′-deoxycytidine, trichostatin A, tideglusib	[73]
Pluripotent stem cells	Reprogramming to hair follicle	Epidermal growth factor, RA, BMP4	[83, 84]

**(b):** 

*In vivo*		
Roles of bioactive molecules	Bioactive molecules	Reference
Regulation of the wound microenvironment Modulation of endogenous stem cell or progenitor cell Stimulation of somatic cell proliferation or repairing their function	Thymosin 4	[96, 97]
	Plerixafor, G-CSF, BIO5192	[98, 99]
	FTY720	[100]
	3,3′-Diindolylmethane	[101]
	Tazarotene	[102]
	Hydroxysafflor yellow A, deferoxamine	[103]
	AES16-2M	[104]
	Lithium chloride	[105]
Reducing fibrosis and treating skin diseases	XAV-939	[106]
	ICG-001	[107]
	SB525334	[108]
	Bacterial cellulose decorated with DAPT-modified gold nanocomposites	[109]
	STA-21	[110]
